# Efficient QCA Exclusive-or and Multiplexer Circuits Based on a Nanoelectronic-Compatible Designing Approach

**DOI:** 10.1155/2014/463967

**Published:** 2014-10-16

**Authors:** Amir Mokhtar Chabi, Samira Sayedsalehi, Shaahin Angizi, Keivan Navi

**Affiliations:** ^1^Department of Computer, Faculty of Engineering, Persian Gulf University, Bushehr 7516913817, Iran; ^2^Faculty of Computer Engineering, Islamic Azad University, South Tehran Branch, Tehran 443511365, Iran; ^3^School of Computer Science, Institute for Research in Fundamental Sciences (IPM), Tehran 1953833511, Iran

## Abstract

Quantum-dot cellular automata (QCA) are a transistorless computation approach which encodes binary information via configuration of charges among quantum dots. The fundamental QCA logic primitives are majority and inverter gates which can be utilized to design various QCA circuits. This study presents a novel approach to designing efficient QCA-based circuits based on Boolean expressions achieved from reconfiguration of five-input and three-input majority gates. Whereas the multiplexer and Exclusive-or are the most important fundamental logical circuits in digital systems, designing efficient and single layer structures without coplanar cross-over wiring is advantageous in QCA technology. In order to demonstrate the efficiency and usefulness of the proposed approach, simple and dense multiplexer and Exclusive-or structures are implemented. The proposed designs have significant improvement in terms of area, complexity, latency, and gate count in comparison to previous designs. The correct logical functionalities of presented structures have been authenticated using QCA designer tool.

## 1. Introduction

Due to current serious exiting challenges in conventional transistor technology, researchers are searching to find an alternative to this technology. Among these new technologies, quantum-dot cellular automata (QCA) are a suitable alternative that offers unique features such as small feature size and ultralow power consumption and can operate at THz frequencies and room temperature [1, 2].

**Figure 1:**
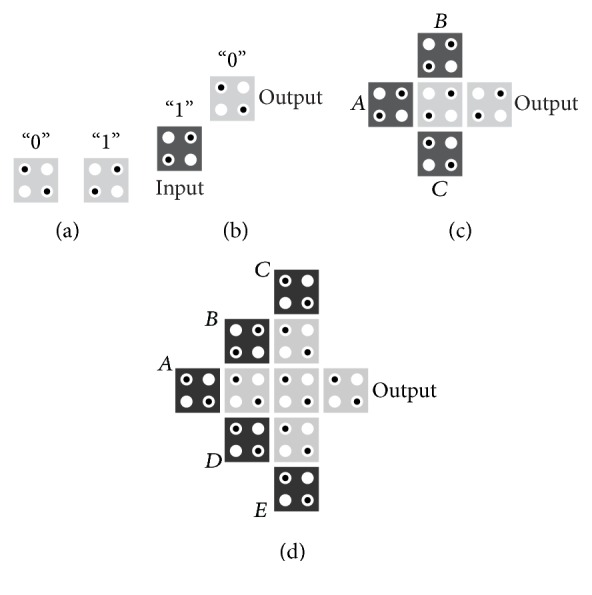
Basic logic cells and gates in QCA: (a) two possible polarizations, (b) inverter, (c) three-input majority gate, and (d) the presented five-input majority gate in [9].

The basic elements in QCA are cells; each cell is composed of two mobile electrons that are located in opposite corners according to columbic energy, resulting in two possible polarizations (*P* = +1, *P* = −1) as shown in Figure 1(a) [3].

Up to this time, many methods for fabrication of QCA basic cells are suggested such as metal island [4], magnetic [5], semiconductor [6], and molecular QCA [7]. As is discussed in [8], metal dot implementations have proven to be the most successful material systems which are based on single-electron transistors' fabrication techniques. The magnetic implementation is firstly proposed by Cowburn's group and extended by the Porod group and the Bokar group. In the semiconductor physical implementation, the Cavendish group of Smith et al. proved QCA operation in GaAs/AlGaAs heterostructures with confining top-gate electrodes and the group of Kern et al. demonstrated a silicon QCA cell by employing an etching technique to form the dots. Furthermore, based on [8], the Fehlner and Lapinte groups have performed successful molecular synthesis in creating molecules that show the essential bistability.

According to the columbic interaction between electrons in neighboring cells, the basic logic gates in QCA circuits (inverter and majority gates) are constructed as shown in Figures 1(b) and 1(c), respectively [9–11]. The logical functions of three-input majority gate and five-input majority gate [9] (Figure 1(d)) are

(1) $$\begin{array}{c} M\left(A,B,C\right)=AB+BC+AC \end{array}$$

(2) M(A,B,C,D,E)=ABC+ABD+ABE+ACD+ACE +ADE+BCD+BCE+BDE+CDE.

This paper presents a new method to design well-organized QCA circuits that reduces the hardware requirements when compared to previously reported circuits. Multiplexer and Exclusive-or are the most significant components in logical systems, so these circuits are optimized based on this method.

The remainder of this paper is arranged as follows. In Section 2, a review on state-of-the-art designs is provided.  Section 3 introduces the new approach to implementing QCA-based structures and proposes efficient and feasible designs for multiplexer and Exclusive-or. In Section 4, we use simulation results obtained from QCADesigner tool to prove the functional correctness of our proposed designs and finally Section 5 concludes the paper.

## 2. State-of-the-Art

As mentioned earlier, the main purpose of this paper is to design two main structures for implementation of various logic circuits, so in this section previous designs are reviewed in order of 2-to-1 multiplexer and Exclusive-or.

### 2.1. Multiplexer

**Figure 2:**
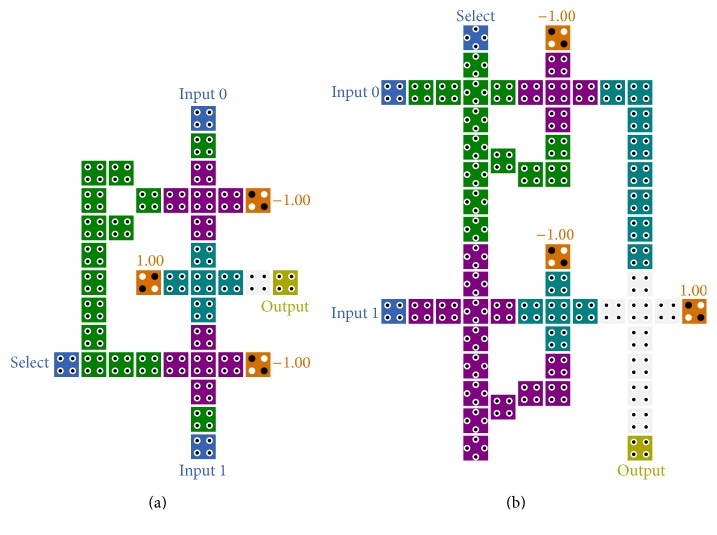
(a) The presented 2-to-1 multiplexer [17]. (b) The presented 2-to-1 multiplexer [16].

Multiplexers have a considerable role in the digital systems which allow us to select one of the input's flows for transmitting to the output. Whereas all the logic functions can be built by multiplexers, implementation of multi-input multiplexer in one layer is a remarkable subject. In conventional implementation of multiplexer, there are several structures that have been introduced in [12–22]; all these designs have tried to present improved structure rather than the other. These designs have been implemented using three three-input majority gates in different ways with different propagation delay and consumed cells according to the form of majority gate's concatenation. One of the best proposed structures in terms of complexity and latency is introduced in [17], as shown in Figure 2(a). An innovative methodology for designing 2-to-1 multiplexer is introduced in [16]. This design has a modular structure that consists of several elementary blocks as illustrated in Figure 2(b). It is noteworthy that the latency of the circuits in large scales has been diminished by utilizing the presented methodology.

These designs have been implemented according to the following equation:

(3) Output=Maj(Maj(Input0,Select¯,0),    Maj(Input1,select,0),1(Input0,Select¯,0),).

### 2.2. Exclusive-or

**Figure 3:**
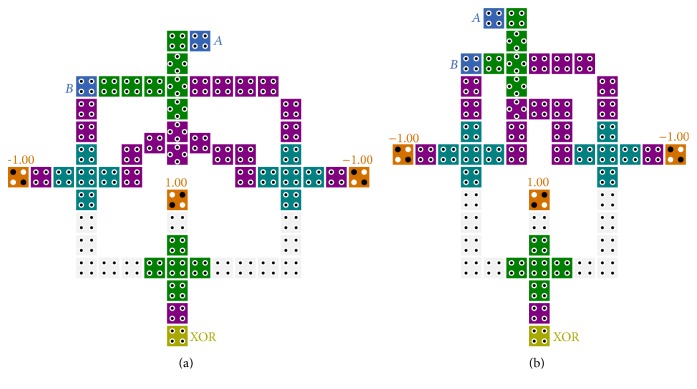
(a) The presented 2-input XOR gate in [23]. (b) The presented 2-input XOR gate using new cell arrangement in [24].

Due to the momentous usage of Exclusive-or component in various tasks such as parity checking and detection and correction mechanism in the receiver and sender units, designing an efficient and high speed Exclusive-or is one of the most important challenges in QCA studies. According to the position of the input signals in the Exclusive-or structure, most of the presented designs are implemented based on the multilayer or coplanar cross-over wiring. In [23, 24], useful implementations of XOR gate are presented which use coplanar cross-over wiring, as demonstrated in Figure 3. These designs have a similar 1.5 clock cycle delay for transmitting input signals to the output.

## 3. Proposed Designing Approach

The main building block of QCA circuits is majority gate and consequently the other logic circuits are implemented based on majority gate networks. In this section, we are going to propose a novel designing approach to implementing QCA logic circuits with least hardware overhead. To overcome this goal, in addition to three-input majority gate, we have employed the five-input majority gate. In this approach, some functional logic circuits are implemented by configuration of five-input majority gate inputs.

**Figure 4:**
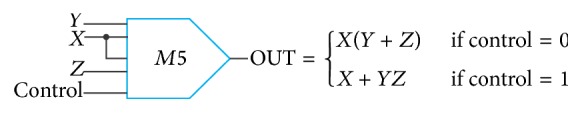
Achieved Boolean expressions based on the proposed method.

As shown in Figure 4, *X*, *Y*, and *Z* are labeled as the main inputs and the control input is labeled as control line. In addition, one of these inputs has twice the effect of the other inputs on five-input majority gate. By setting control line to “1” logic value, the Boolean function *X* + *YZ* is obtained. Furthermore, logical function *X*(*Y* + *Z*) can be achieved by changing the value of the control line to “0.”

By using the five-input majority function (2), we get the following equations:

(4) $$\begin{array}{c} M5\left(Y,X,X,Z,0\right)=YX+YXZ+YXZ+XZ=X\left(Y+Z\right), \end{array}$$

(5) M5(Y,X,X,Z,1)=YX+YXZ+YX+YX+YX+YZ +XZ+X+XZ+XZ=X+YZ.

It is worth mentioning that these functions need only one five-input majority gate for implementation. However, these functions are designed with two three-input majority gates in the conventional method.

From the achieved equations, we can conclude that most of the combinational and sequential circuits can be constructed by assigning proper functions or fixed values to their parameters. The next section provides implementation steps of multiplexer and Exclusive-or circuits based on this method.

### 3.1. Multiplexer Design

**Figure 5:**
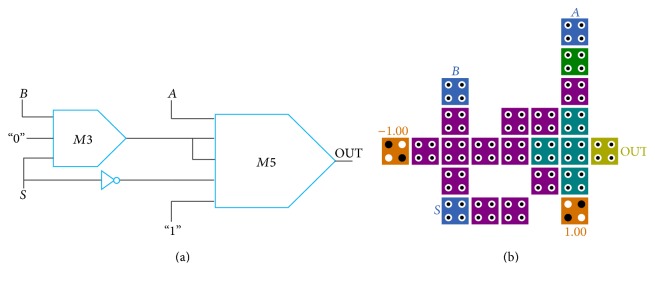
(a) Schematic of the proposed 2-to-1 multiplexer based on new approach. (b) QCA implementation of 2-to-1 multiplexer.

As noted above, a feasible design for 2-to-1 multiplexer can be obtained by utilizing the proposed novel method. According to the 2-to-1 multiplexer function, the inputs of logical function *X* + *YZ* should be changed as in Figure 5(a).

The logic function *B* · *S* should be fed to input *X* and also the inputs *A* and $\bar{S}$ should be fed to the inputs *Y* and *Z*, respectively. For implementation of logical function *BS*, a three-input majority gate has been used. It is to be noted that only two majority gates and one inverter gate are used for implementing this structure.

By applying this method, the equation of 2-to-1 multiplexer is defined as follows:

(6) $$\begin{array}{c} M5\left(A,M3\left(B,0,S\right),M3\left(B,0,S\right),\bar{S},1\right), \end{array}$$

(7) $$\begin{array}{c} M5\left(A,BS,BS,\bar{S},1\right)=A\bar{S}+BS. \end{array}$$

To clarify the correct functionality of the proposed design in detail, the truth table of proposed circuit is shown in Table 1 with three output columns. The first column presents eight possible combinations of three input cells (*S*, *A*, and *B*). The second column demonstrates the output of three-input majority gate which produces the logical function *B* · *S*. The summation of five-input majority gate inputs is shown in the third column and the last column illustrates the main output of the proposed 2-to-1 multiplexer circuit.

As it is obvious in Figure 5(b), the latency of proposed multiplexer is 0.75 clock cycle, so this design is the fastest in comparison to previous mentioned designs.

### 3.2. Exclusive-or Design

**Figure 6:**
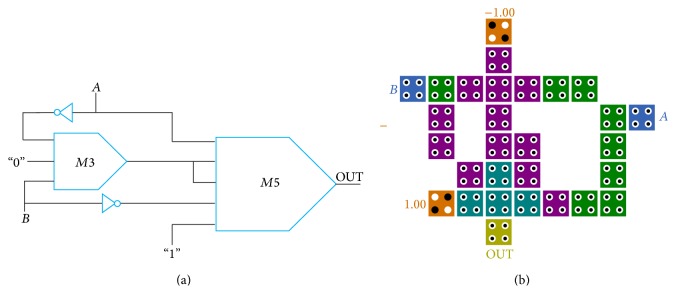
(a) Schematic of the proposed two-input Exclusive-or based on new approach. (b) QCA implementation of two-input Exclusive-or.

In this section, we propose our new high speed and single layer two-input XOR gate using the *X* + *YZ* equation mentioned above. The applied equation which is achieved using the majority gate functions is shown in (9). As illustrated in Figure 6(a), the output of three-input majority gate ($\bar{A}B$) with twice the effect and inverse of *B* signal and *A* signal are assigned to the five-input majority gate in the similar clocking zone. Consequently, the Exclusive-or of *A* and *B* signals is produced in the next clocking zone. Considering the similar procedure, we get the equation of two-input XOR gate as follows:

(8) $$\begin{array}{c} M5\left(A,M3\left(\bar{A},0,B\right),M3\left(\bar{A},0,B\right),\bar{B},1\right), \end{array}$$

(9) $$\begin{array}{c} M5\left(A,\bar{A}B,\bar{A}B,\bar{B},1\right)=\bar{A}B+A\bar{B}. \end{array}$$

**Figure 7:**
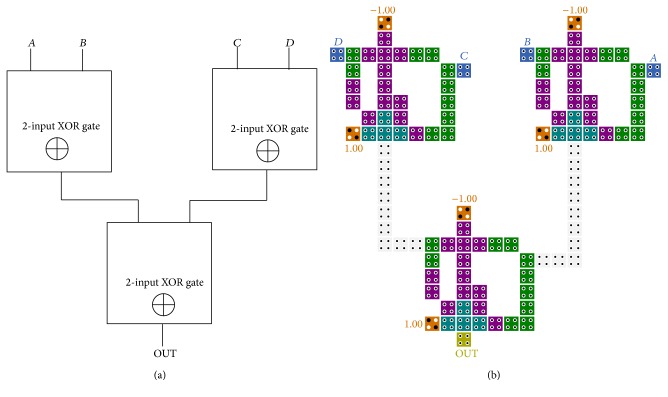
(a) Block diagram of four-input XOR gate. (b) QCA layout of four-input XOR gate based on cascading of the proposed XOR gate in Figure 6(b).

The propagation delay of the presented two-input XOR gate is 0.75 clock cycle with huge reduction in cell counts and area occupation. The significant contribution of this design is implemented in signal layer without using rotated cells in comparison to previous designs. This structure can be expanded to the larger scale by cascading several two-input XOR gates. As example, the schematic of four-input XOR gate and its QCA implementation are shown in Figure 7.

## 4. Simulation Results

QCADesigner is a well-known simulation tool generally expanded for evaluating QCA logic circuits; this tool has two different simulation engines which are called bistable approximation and coherence vector. The bistable approximation engine calculates state of a single cell using a time-independent approach with kink energy formula that calculates cost of two cells having opposite polarizations, so simulation time in this engine is reduced. The coherence vector model considers the time-dependent state of a cell in interaction with the other cells through the same kink energy formula [25–27].

**Figure 8:**
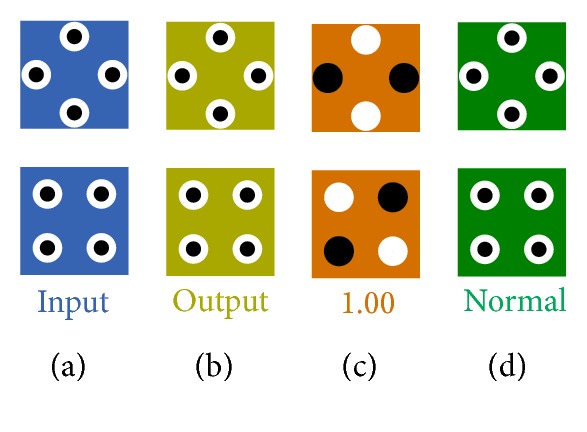
QCADesigner cells: (a) input cell, (b) output cell, (c) fixed polarization cell, and (d) normal cell.

In QCADesigner software, each single cell (standard, rotated) can act in four modes (input, output, fixed, and normal) which is shown in Figure 8.

In this section, correct functionality of the proposed structures is authenticated using QCADesigner tool version 2.0.3 [25]. Each one of the circuits is examined under both simulation engines (bistable approximation and coherence vector) and similar outcomes are achieved. Tables 2 and 3 illustrate the applied parameters in bistable approximation and coherence vector simulation engines, respectively.

**Figure 9:**
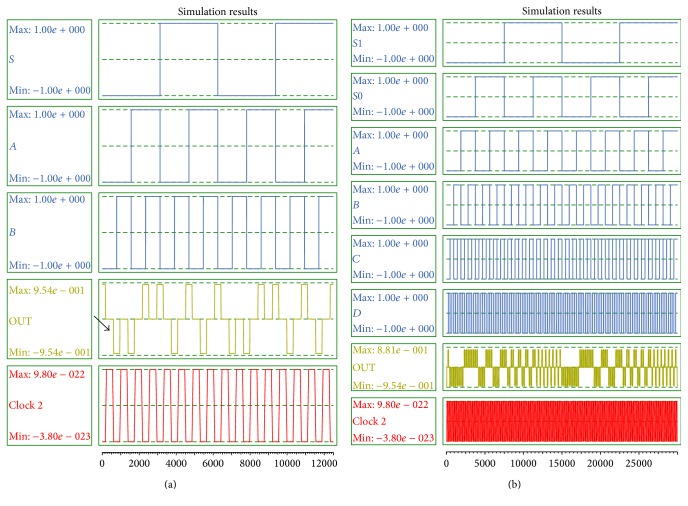
(a) Simulation result of the proposed 2-to-1 multiplexer. (b) Simulation result of 4-to-1 multiplexer.

The analysis of output waveforms verifies the accuracy and efficiency of presented designs in comparison to state-of-the-art designs. The simulation results of proposed 2-to-1 multiplexer and 4-to-1 multiplexer are illustrated in Figures 9(a) and 9(b), respectively. As is clear in Figure 9(a), the first meaningful output appears in 0.75 clock cycle.

**Figure 10:**
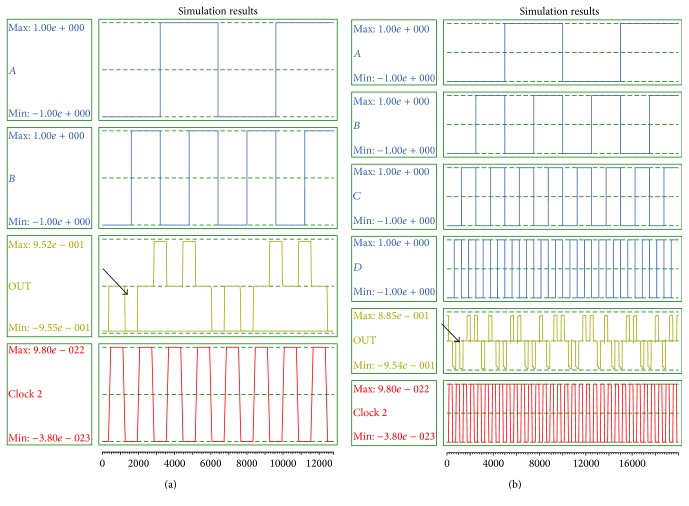
(a) Simulation result of the proposed two-input XOR gate. (b) Simulation result of four-input XOR gate.

In Figures 10(a) and 10(b), simulation results of two-input and four-input Exclusive-or circuits are authenticated. Latency of the two-input and four-input XOR gates is 0.75 and 2.75 clock cycle, respectively.

Table 4 comprises the previous works in 2-to-1 and 4-to-1 multiplexer's designs with the proposed multiplexers in terms of hardware requirement and latency.

According to Table 5, it can be concluded that this proposed design results in significant improvements in gate count, area, cell count, and latency. The comparison results of Exclusive-or circuits are illustrated in Table 6. Based on the obtained results, it can be concluded that employing the proposed approach in QCA circuits leads to a considerable optimization in cell count, occupation area, and propagation delay.

## 5. Conclusion

In this paper, the new approach to implementation of QCA-based circuit was introduced. This method is based on the new configuration of five-input majority gate that led to achieve significant Boolean function such as *X* + *Y* · *Z*. It is expected that the novel method presented in this paper will produce efficient QCA-based logical circuits such as multiplexer and Exclusive-or. These proposed circuits surpass previous designs in terms of gate count, area, cell count, and latency. Furthermore, the great advantage of the presented approach is that it leads to implementation of these structures in single layer without any cross-over wiring.

## Figures and Tables

**Table 1:** Truth table of functionality of proposed circuit.

*S*	*A*	*B*	*X* = *S* · *B*	2*X* + *Y* + *Z* + 1	OUT

0	0	0	0	2	0
0	0	1	0	2	0
0	1	0	0	3	1
0	1	1	0	3	1
1	0	0	0	1	0
1	0	1	1	3	1
1	1	0	0	2	0
1	1	1	1	4	1

**Table 2:** Bistable approximation parameters model.

Parameter	Value
Cell size	18 nm
Number of samples	50000
Convergence tolerance	0.001000
Radius of effect	65.000000 nm
Relative permittivity	12.900000
Clock low	3.800000*e* − 023 J
Clock high	9.800000*e* − 022 J
Clock shift	0
Clock amplitude factor	2.000000
Layer separation	11.500000
Maximum iterations per sample	100

**Table 3:** Coherence vector parameters model.

Parameter	Value
Temperature	1.000000 K
Relaxation time	4.1356675*e* − 14 s
Time step	1.000000*e* − 016 s
Total simulation time	7.000000*e* − 011 s
Clock high	9.800000*e* − 022 J
Clock low	3.800000*e* − 023 J
Clock shift	0.000000*e* + 000
Clock amplitude factor	2.000000
Radius of effect	80.000000 nm
Relative permittivity	12.900000
Layer separation	11.500000 nm

**Table 4:** Comparison results of the presented multiplexers.

	Circuit	Gate count (majority gate + inverter)	Area (*μ*m^2^)	Cell count	Latency (clock)	Cross-over type
2 : 1 multiplexer	[12]	4	0.08	46	1	Multilayer
	[13]	4	0.06	36	1	Multilayer
	[14]	3	0.07	54	1	Coplanar
	[15]	3	0.09	60	1	Coplanar (rotated cells)
	[16]	3	0.07	56	1	Coplanar
	[17]	4	0.05	38	1	Not required
	[18]	5	0.02	23	3/4	Not required
	The proposed	3	0.03	23	3/4	Not required

4 : 1 multiplexer	[19]	15	0.25	246	1 + 1/4	Multilayer
	[20]	18	0.25	124	2	Not Required
	[21]	11	0.15	154	1	Multilayer
	[14]	9	0.24	159	2 + 1/4	Coplanar
	[15]	9	0.72	456	2 + 3/4	Coplanar
	[16]	11	0.25	215	1 + 1/2	Coplanar
	[22]	14	0.08	73	1	Coplanar
	The proposed	9	0.11	82	1 + 3/4	Not required

**Table 5:** The proposed multiplexer improvements in comparison to other previous designs.

Improvement	Circuit	Gate count (majority gate + inverter)	Area	Cell count	Latency
2 : 1 multiplexer	[12]	25%	62%	50%	25%
	[13]	25%	50%	36%	25%
	[14]	0%	57%	57%	25%
	[15]	0%	66%	61%	25%
	[16]	0%	57%	59%	25%
	[17]	25%	40%	39%	25%
	[18]	40%	−50%	0%	0%

4 : 1 multiplexer	[19]	40%	56%	66%	−28%
	[20]	50%	56%	33%	12%
	[21]	18%	26%	46%	−42%
	[14]	0%	54%	48%	22%
	[15]	0%	84%	82%	36%
	[16]	18%	56%	61%	−16%
	[22]	35%	−37%	−12%	−75%

**Table 6:** Comparison results of presented Exclusive-or designs.

Circuit	Gate count	Area (*μ*m^2^)	Cell count	Latency (Clock)	Cross-over type
XOR 2 [23]	5	0.09	60	1.5	Coplanar (rotated cells)
XOR 2 [24]	5	0.08	54	1.5	Coplanar (rotated cells)
The proposed XOR 2	4	0.03	29	0.75	Not required
The proposed XOR 4	12	0.19	106	1.75	Not required
The proposed XOR 8	28	0.60	269	2.75	Not required
